# Visuo-acoustic stimulation that helps you to relax: A virtual reality setup for patients in the intensive care unit

**DOI:** 10.1038/s41598-017-13153-1

**Published:** 2017-10-16

**Authors:** Stephan M. Gerber, Marie-Madlen Jeitziner, Patric Wyss, Alvin Chesham, Prabitha Urwyler, René M. Müri, Stephan M. Jakob, Tobias Nef

**Affiliations:** 1 Gerontechnology & Rehabilitation Group, University of Bern, Bern, Switzerland; 2 Department of Intensive Care Medicine, University Hospital Bern (Inselspital), University of Bern, Bern, Switzerland; 3 University Hospital of Old Age Psychiatry, University of Bern, Bern, Switzerland; 4 Department of Neurology, University Neurorehabilitation, University Hospital Bern (Inselspital), University of Bern, Bern, Switzerland; 5 ARTORG Center for Biomedical Engineering Research, University of Bern, Bern, Switzerland; 6 Neurology and Neurorehabilitation Center, Luzerner Kantonsspital, Luzern, Switzerland

## Abstract

After prolonged stay in an intensive care unit (ICU) patients often complain about cognitive impairments that affect health-related quality of life after discharge. The aim of this proof-of-concept study was to test the feasibility and effects of controlled visual and acoustic stimulation in a virtual reality (VR) setup in the ICU. The VR setup consisted of a head-mounted display in combination with an eye tracker and sensors to assess vital signs. The stimulation consisted of videos featuring natural scenes and was tested in 37 healthy participants in the ICU. The VR stimulation led to a reduction of heart rate (p = 0. 049) and blood pressure (p = 0.044). Fixation/saccade ratio (p < 0.001) was increased when a visual target was presented superimposed on the videos (reduced search activity), reflecting enhanced visual processing. Overall, the VR stimulation had a relaxing effect as shown in vital markers of physical stress and participants explored less when attending the target. Our study indicates that VR stimulation in ICU settings is feasible and beneficial for critically ill patients.

## Introduction

After prolonged stay in intensive care units (ICU), patients often complain about cognitive impairment which can lead to a reduction of health-related quality of life as compared to before ICU admission^1,2^. Such patients may initially have suffered from severe infection, multiple trauma injuries, heart failure and other diseases leading to multiple organ failure that requires intensive or emergency treatment. In recent years, intensive care medicine has seen a culture change in that the focus on improving survival rates has been expanded to include long-term health-related quality of life after discharge^3^. Health disorders seen in patients, collectively referred to as post intensive care syndrome, include persistent impairments across physical, psychiatric and cognitive domains^4^. Long-term cognitive impairment affects between 50% and 75% of patients, independent of cognitive status prior to admission, involvement of brain injury and age of the patients^4–7^. Long-term cognitive impairment frequently manifests first as acute brain dysfunction (delirium and coma) when patients are being treated for critical illness in the ICU^8^. Thereafter, long-term cognitive impairment is known to affect neurocognitive functions such as information processing, concentration, attention, learning and memory as well as executive functions^6,9^.

Current treatment modalities that address cognitive impairment in ICU survivors include preventive and remedial measures as well as drug therapy during and after the course of the ICU stay. Preventive strategies, on the one hand, are aimed at reducing various risk factors for cognitive dysfunction and psychological morbidity during ICU stay. These include early physical rehabilitation, sleep promotion, minimization of use of sedatives and psychological support (e.g. counselling)^10^. These approaches have been reinforced with findings from studies that have shown that early mobilization and physical training, and choice of sedation can indeed reduce cognitive impairment^5^. Furthermore, the “ABCDE” strategy (awakening, breathing, choice of sedatives, daily delirium monitoring, and early mobilization) has been specifically targeted at reducing sedation and delirium that are suspected to cause post-ICU cognitive impairment^11^. While these strategies were shown to reduce delirium, it has not yet been demonstrated that they actually prevent cognitive impairment^4^. Remedial strategies, on the other hand, use cognitive interventions aimed at retraining impaired cognitive functions and thus improve everyday functions. Several authors have highlighted the potential benefit of early cognitive and physical therapy during the ICU stay. However, most studies were conducted in critically ill patients after the stay in the ICU^5,6,12,13^ and therefore evidence from interventional studies in critically ill patients remains scarce particular during ICU stay^4^. It is assumed that early onset of intervention will have the biggest impact on later cognitive and psychological morbidity by both preventing cognitive impairments from developing and improving cognitive functions after discharge^4,10^.

A critical factor in critically ill patients is the typical ICU environment characterized by constant exposure to unpatterned stimuli such as light, noise, lack of daily living routine and limited mobility^14^. Patients are exposed to the extremes of both sensory overload and sensory deprivation and are at risk of suffering alterations in sensory perception that may result in cognitive dysfunction^15^. Sensory overload can be produced by stimulation at above normal levels with continuous and excessive unfamiliar sensory input^16^. Sensory deprivation is caused by reduced amounts or variability of stimulation with decreased or monotonous, unpatterned and meaningless sensory input^17^. It is the resulting combination of both stimulus overload and simultaneous stimulus deprivation that is believed to lead to a loss of differentiated perception, orientation and cognitive and attentional fatigue in critically ill patients^18–20^. This perception disorder is further reinforced by medication and therapeutic measures such as analgesia or sedation. These environmental stressors also cause considerable psycho-physiological stress, especially in older patients.

Chen *et al*. and Turon *et al*. suggested that virtual reality (VR) technology might be useful to stimulate critically ill patients during ICU stay^21,22^. VR using head-mounted displays (HMD) seems to be a promising tool to cognitively stimulate critically ill patients in a safe way with the ability to fully control virtual environments that users can interact with and immerse themselves in. Thus, virtual-reality based cognitive stimulation can be used for improving cognitive functioning in critically ill patients while at the same time preventing stimulus overload by isolating the patient from disturbing external visual and acoustic input. To avoid both sensory overload and sensory deprivation, we selected natural restorative environments with a neutral content.

In VR a distinction is made between virtual environments (VE) and virtual worlds (VW). VE use two or three-dimensional environments that include characters and objects that users can see, hear and interact with in a realistic manner in real time. VW are persistent and social worlds where users can interact with other users using an artificial avatar^23,24^. Furthermore, one can distinguish in VR between active mode navigation where users interact in real-time with the VE and thus have a sense of control over the scenario (e.g. controlling of an avatar), whereas in passive mode navigation (e.g. watching immersive videos) they have not. In both cases VR permits the users to experience VE that would otherwise be difficult or even impossible to implement in real life. To achieve this goal, two components are needed: an appropriate algorithm to render the moving objects and the surrounding environment and a physical device (e.g. HMD) to present the images to the user^25^. Thus, one of the core elements of VR is the interface between the user and the virtual world. Furthermore, VR environments need to be carefully designed in order to minimize sensory conflicts (e.g. between visual and vestibular system) to avoid cybersickness^26,27^.

To our knowledge, there is currently no data on the use of VR for cognitive stimulation in critically ill patients during an ICU stay. One challenge when using VR stimulation in critically ill patients is to select a suitable virtual scenario and ensure that it has a relaxing effect and engages the patient’s attention^28^. An established line of research has suggested that visual exposure to natural environments (e.g. landscapes, vegetation, and water) has protective effects against environmental stressors and restores physiological, emotional and attentional functions^29–31^. These restorative effects have been explained within two complementary theories: The Stress Recovery Theory posits that natural environments support positive changes in emotional states and recovery from psycho-physiological stress through a relaxing effect in the parasympathetic system^32^. This is relevant since the ICU environment can create considerable stress in patients that affects well-being and recovery. Furthermore, cognitive or attentional capacity, can become fatigued in stressful environments^33^. This is crucial for critically ill patients facing stimulus overload and high demand on cognitive capacity in ICU settings that increase the risk for mental fatigue and stress response. The Attention Restoration Theory states that exposure to natural environments is less cognitively demanding and promotes a sense of being away, thus enabling attentional capacity to rest and be restored^30,33,34^.

In this study, we propose to use video oculography (integrated into the HMD) to measure the visual exploration behaviour (e.g. where the patient “looks at” and whether the eyes are open). To measure participant’s reactions to the VR Stimulation, we use vital parameters that are routinely recorded in ICU settings (e.g. heart rate, blood pressure etc.), while presenting three different nature VR videos, i.e. landscape, water worlds and animals.

We hypothesise that stimulation with the three nature VR videos has a relaxing effect (measured via vital markers of physical stress^35^), the visual explorative behaviour follows the scene contents (e.g. participants look at a visual target, detailed description in method section), creates a sense of immersion (measured by an 5 scale questionnaire) and the technical implementation meets the hygiene and security requirements for the use in critically ill patients. In the present study, feasibility and proof of concept of VR Stimulation was investigated in 37 healthy participants.

## Results

### Descriptive analysis of pooled vital sign- and oculomotor measurements

**Figure 1:**
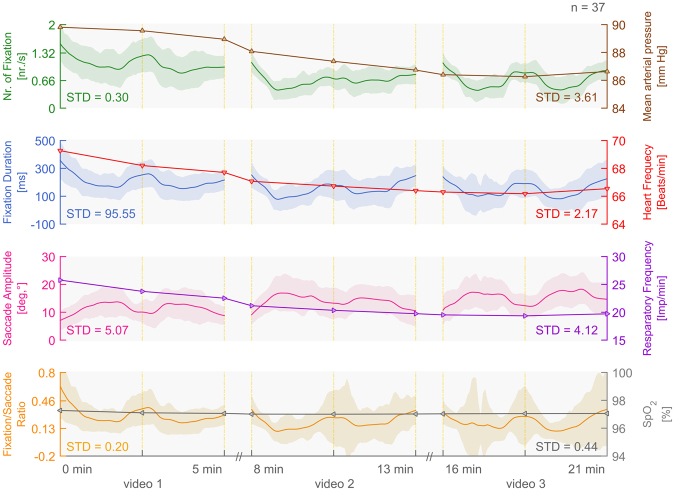
Vital parameters pooled with oculomotor data of healthy participants (n = 37, age M = 48 years, SD = 17). The oculomotor data (fixation duration, number of fixations, saccade amplitudes and fixation/saccade ratio) is based on moving window calculations, whereas the vital sign measurements (MAP, HF, RF and SpO_2_) were sampled at each data points separately.

A total of 37 (23 female and 14 male) healthy younger and older adults between the ages of 20 and 85 (M = 48, SD = 17) participated in this study. In the initial measurement before the recovery phase (i.e. when the participants were lying on the bed 10 minutes before the start of the stimulation) the mean arterial pressure (MAP) was M = 86.8 mm Hg (SD = 23.1), the heart frequency (HF) M = 68.7 beats/min (SD = 21.1) and the peripheral capillary oxygen saturation (SpO_2_) M = 92.3% (SD 22.4). The respiratory frequency (RF) could not be measured accurately since participants moved or the ECG was not placed properly at the beginning. The vital sign measurements MAP (M = 87.67 mm Hg, SD = 1.67), HF (M = 67.02 beats/min, SD = 1.08) and RF (M = 21.08 Imp/min, SD = 2.29) showed a significant decrease during presentation of the three VR scenarios, indicating a negative time effect. The SpO_2_ (M = 97.05%, SD = 0.09) to measure deterioration of participant’s condition remained constant (Fig. 1). However, oculomotor data did not change much across the VR stimulation. The fixation duration per second across stimulations was M = 170.15 ms (SD = 55.42), the number of fixations M = 0.81 fixations (SD = 0.25), the saccade amplitude M = 13.74 degrees (SD = 2.60) and fixation/saccade ratio M = 0.24 (SD = 0.09).

Identification of the visual target that appeared at the beginning, middle, and end of each video was reflected in peaks of number and duration of fixations and fixation saccade ratios, whereas, reversely, the saccade amplitudes dipped at the start, in the middle, and at the end of each video.

### Effect of time and ratings on vital signs

Data analysis using linear mixed effect models (LMEMs) to generalize the findings revealed a significant reduction of vital signs (HF, t (276) = −2.704, p = 0.007, RF t (276) = −2.020, p = 0.044 and MAP, t (276) = −1.981, p = 0.049) during the course of the stimulation (negative time effect) across all participants, except in SpO_2_ (t (277) = −1.377, p = 0.170) which did not significantly decrease during the stimulation. However, in HF, there was a significant difference in slope between participants (inter-individual difference) during the course of the study. For RF and MAP the time effect was significantly higher for participants with higher baseline vital sign measurements. Overall, there was no significant interaction between time and age (fixed interaction effect) except for MAP (t (276) = −2.149, p = 0.033) indicating an increasing reduction of vital signs during the course of the stimulation (negative time effect) with increasing age. There was no significant effect of video type on any of the vital signs measurements. Furthermore, none of the questionnaire scales demonstrated a significant influence on vital signs. Given the high relationship between time and video type (given by the design), the variable video type was not removed from the LMEMs.

### Effect of target presentation and time in oculomotor data

Generalized findings using LMEM analysis as shown in Table 1 across all participants revealed that oculomotor data during stimulation stayed more or less constant (time effect), indicated by the small effect in number of fixations, fixation duration, saccade amplitude and fixation/saccade ratio. Therefore, the time effect in oculomotor data is negligible. However, there was a strong significant effect of target presence on the number of fixations, fixation duration, saccade amplitude and fixation/saccade ratio.

**Table 1:** Effect of time and target in oculomotor data by using LMEM analysis.

	Nr. of fixation	Fixation duration	Saccade Amplitude	Fixation/saccade ratio
*Time*	β = −2.511e-4	β = −0.0479	β = 1.372e-03	β = −1.033e-04
	t (1781611) = −134.22	t (1781611) = −69.47	t (1781611) = 39.65	t (1781611) = −59.41
	p < 0.001	p < 0.001	p < 0.001	p < 0.001
*Target*	β = 0.263	β = 81.29	β = −3.42	β = 0.128
	t (1781611) = 814.07	t (1781611) = 682.22	t (1781611) = −571,88	t (1781611) = 426.85
	p < 0.001	p < 0.001	p < 0.001	p < 0.001

Given the large set of oculomotor data, it was difficult to interpret the p-values (p-value inflation). Therefore, the LMEM accuracy (effect size) was assessed by examining the marginal (${R}_{{LMM}(m)}^{2}$) and conditional (${R}_{{LMM}(c)}^{2}$)R^2^ as proposed by Nakagawa *et al*. and Nuthmann *et al*.^36–38^. The goodness of fit of the full model revealed that the proportion of the variance explained (${R}_{{LMM}(m)}^{2}$) by the fixed components was in average M = 18.89%, Min = 9.04%, Max = 32.32% while the whole LMEM, including the random and fixed components (${R}_{{LMM}(c)}^{2}$) was in average M = 52.64%, Min = 36.77%, Max = 71.08%.

### Analysis of immersion, presence, sickness and usability

All average ratings of the five scales of the questionnaire by using t-tests (Table 2) were significantly above (i.e. usability, immersion, presence, realism, involvement) or below (i.e. sickness, oculomotor problems, disorientation) the midpoint of the scale (i.e. 2.5). The usability rating for the whole system was high and close to the maximum score of the questionnaire scale (max = 5). Sickness, oculomotor problems, and disorientation ratings were relatively low and close to the minimum score of the scale (min = 1). Immersion, presence, realism and involvement ratings were average, ranging around the midpoint of the scale with relatively large standard deviations.

**Table 2:** Ratings of the questionnaire by using t-tests. The items had a five-point rating scale between one and five.

	Questionnaire-Nr.	M	SD	t(36)	p
*Usability*	7–9	4.5	0.6	20.44	<0.001
*Sickness*	10–12, 15	1.16	0.4	−18.10	<0.001
*Oculomotor problems*	13–14	1.35	0.5	−14.03	<0.001
*Disorientation*	16	1.16	0.5	−16.25	<0.001
*Immersion*	1	3.6	1.2	5.6	<0.001
*Presence*	2	3.11	1.1	3.36	<0.001
*Realism*	5	2.92	1.38	1.84	<0.001
*Involvement*	3–4, 6	3.08	0.8	4.61	<0.001

## Discussion

### Principal Results

In this study, we investigated the feasibility and proof of concept of VR stimulation in healthy participants in the ICU. In line with our first hypothesis, we found that exposure to virtual nature environments produced a relaxing effect as measured by vital signs. Second, there was no indication of fatigue in visual exploration during VR stimulation and target stimulus presentation affected visual exploration behavior (visual search vs. visual processing). Third, the VR scenario was well tolerated by the participants and allowed them to fully immerse themselves within the video content. Finally, we showed that a commercially available VR setup can be adapted to comply with hospital hygiene and safety requirements for use in critically ill patients.

### Vital signs

The first main finding was that all vital sing measurements (heart frequency, respiratory frequency and mean arterial blood pressure) except blood oxygen saturation significantly decreased during the course of the VR stimulation. This can be interpreted in away, that VR stimulation using virtual nature environments was successful in reducing measures of physiological stress. Furthermore, the relaxing effect was independent of age in heart frequency and respiratory frequency. However, mean arterial blood pressure showed an increase of the relaxing effect in older participants. Since blood oxygen saturation increase did not reach significance, we find reason to assume that the participants’ condition was not deteriorated during VR stimulation.

Chirico, *et al*. and Joye and Bolderdijk *et al*. have shown that the exposure to VR nature environments can trigger specific psychophysiological patterns of parasympathetic activation, induce strong emotions and that the emotional reactions increases with the level of immersion. Emotions evoked by means of VR have been successfully implemented in treatments for anxiety and phobia stress inoculation^39–41^. Our finding of a relaxing effect could be due to the positive emotions of the immersive nature videos. This also matches the Stress Recovery Theory suggesting that exposure to virtual nature environments has a restorative influence that can reduce physiological stress responses and increase well-being^32,42^. This is further confirmed by recent studies that showed that stress recovery can be facilitated using immersive virtual natural scenes combined with sounds^29,43^.

### Occulomotor data

The second main finding confirms that stimulation using natural videos was successful in capturing participants’ attention. The absence of a time effect in the oculomotor data indicates the absence of mental fatigue during VR stimulation which coincide with findings in literature^44,45^. In line with the Attention Restoration Theory, this might suggest that the virtual nature environments used in our study allowed participants to rest and restore attentional capacity^34^.

The fixation/saccade ratio represents the ratio between information processing and search activity, where a higher ratio reflects more processing and less search activity, while a low ratio reflects more search activity and less processing^46^. From this it is evident that participants were visually exploring the videos less when the target stimulus was present as compared to when the target stimulus was not present.

The goodness of fit of the LMEM models of the oculomotor data compared to a recent study reporting similar models^36^, indicating a very good fit of the models to the presented data, which further confirms the significance of the findings of oculomotor data.

Given that this study examined visual exploration using an HMD, it is difficult to relate our findings to those from previous studies that consistently used desktop screens. While the latter offers a field of view of 48 by 27 deg., the HMD in this study had a larger field of view of 95 by 106 deg. This is crucial since it was shown that the larger the visual field size, the lower the fixation durations and the larger the fixation amplitudes are^47^. Hence, it is hardly not surprising that the magnitude of fixation durations (170.2 ms) was almost half as high and saccade amplitude (13.7 deg.) twice as high when compared to the findings of a previous study during dynamic viewing of natural scenes (326 ms, 7.2 deg.)^48^. The larger visual field size in VR setups does not only more closely matches natural visual exploration behaviour, it also provides participants with a higher sense of immersion. Therefore, the use of HMD based VR stimulation, in line with relevant literature, is suitable for critically ill patients^49^.

### Questionnaire

The questionnaire responses, measured by a 5 – point scale (range one to five), clearly indicate that the HMD-based VR stimulation is well accepted and appreciated by the participants as shown in the high usability score (M = 4.5, SD = 0.6). Immersion (M = 3.60, SD = 1.2) and presence (M = 3.11, SD = 1.1) were also at a high level, and thus participants were not aware of their surrounding and could focus on looking at the VR scenario. We believe that participants were well isolated from the meaningless and unpatterned stimuli such as noise, light and bustle of the ICU environment. Symptoms of cybersickness (M = 1.16, SD = 0.4) were rated low on the questionnaire scale and are therefore negligible. The presented results are an important prerequisite for the future use of the implemented VR setup in critically ill patients.

### Technical implementation

Overall, a beyond state of the art mobile HMD-based VR setup to stimulate participants in the ICU was successfully implemented. The setup was running solid and no data loss or any complication on participants occurred. Furthermore, the system fulfilled all standard medical hygiene and safety requirements for critically ill patients.

A number of studies have shown cognitive benefits of VR-based rehabilitation in patients with brain-injury and that VR was efficient and well tolerated in acute inpatient medical settings (e.g. pain management, eating disorders, cognitive and motor rehabilitation)^50,51^. To the best of our knowledge, however, no study has investigated the cognitive benefits of VR rehabilitation in critically ill patients during their stay in the ICU. We believe that VR stimulation will be beneficial for critically ill patients with neurological impairments and will become essential in the future since it has the potential for alternative and new trainings. The advantage of VR in the ICU is that it allows the patient to interact with the virtual environment via HMD directly at the bed with little or no support. In addition, VR offers the possibility to simulate many different real or imaginary scenes with an infinite number of targets and distractors while providing a safe and consistent environment^52^. Therefore, the stimulation can be easily adapted to critically ill patients. Typically, this opportunity is not possible for real-world exercises.

### Limitations

To rule out that the relaxing effect in vital signs was not simply a result from having the participant lying on a bed, we had the participants lying on the bed ten minutes prior to the start of the VR stimulation. In addition, the 2D videos were played in the VE without stereoscopic depth and active mode navigation. Nevertheless, the use of 3D VE and active mode navigation could even increase immersion and thus the relaxing effect.

In this study, we did not control for self-reported ratings of participants’ affect and emotional response to the VR stimulation. Even though induction of positive and reduction of stress-related and negative emotions plays an important role in the stress recovery effect of natural environments, this study focused on the effects of natural virtual environments on measures of physiological stress and visual exploration behaviour. Despite the very promising findings it remains unclear whether the findings can be generalized to critically ill patients.

### Guidelines for future VR studies in the ICU

In order for patients to profit from the restorative immersive VR stimulation, the VR stimulation should be intuitive to use and the patients’ Richmond Agitation-Sedation Scale (RASS), a measure of sedation related to consciousness, should not be lower than minus two (light sedation, reaction to voice with eye contact)^53^. Furthermore, the setup should be audited by a notified hospital body regarding hygiene and medical eligibility requirements and declared secure to use in critically ill patients. This requires that all computer fans must be protected with a filter, that the system has a potential equalisation, that no current can be transmitted to the patient and that all parts in contact with the patient are disinfectable. In order that patients can fully immerse themselves, reduce stress and restore their visual attention, we hypothesize that natural VEs, VWs or stereoscopic videos must be fascinating, rich and harmonious as well as promote a sense of being away. To analyse the effect of VR stimulation on cognition in critically ill patients, cognitive tests should be administered prior to ICU admission, during the ICU stay and after discharge. Furthermore, neurological tests and medication, conscious state, delirium and duration of the stimulation should be recorded. Finally, future results should be compared to the current study with healthy participants.

### Conclusion

VR stimulation had a relaxing effect as shown in vital markers of physical stress and was well tolerated by the participants. Furthermore, the VR stimulation was successful in engaging visual attention (exploration and processing) and allowed participants to fully immerse themselves in the virtual environment. This study indicates that VR stimulation has the potential to reduce psycho-physiological stress and to restore cognitive and attentional capacities in critically ill patients. Furthermore, the implemented HMD-based VR setup using visual and acoustic stimulation is feasible and has great potential for cognitive stimulation in critically ill patients.

## Methods

### Participant recruitment and Demographics

The study design was carried out in accordance with the current version of the Declaration of Helsinki and approved by the Ethics Committee of the Canton of Bern, Switzerland (KEK-Nr. 148/15). The study participants were recruited via email advertisement among the Seniors University of Bern and local research groups. All procedures related to the study were explained to the participants and a written informed consent was obtained prior to participation. The main exclusion criteria were age <18 years, visual- and auditory impairments and any long-term medication which could influence vital signs (e.g. blood pressure medication).

### Apparatus

**Figure 2:**
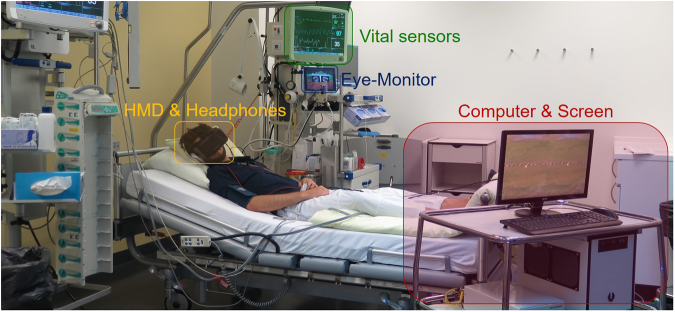
Participant lying on the bed in the ICU during stimulation, including the whole setup.

The study was conducted in a fully equipped 2 bed ICU cubicle. The VR setup to measure and stimulate the participants consisted of two main parts: Firstly, a custom-built desktop computer and a head-mounted display (Oculus Rift DK2 virtual glass) with a built-in SMI Oculus eye tracker system. Secondly, a vital signs monitoring system (Carescape Monitor B650, GE Healthcare, Little Chalfont, UK) was used to record vital parameters such as peripheral capillary oxygen saturation, mean arterial pressure, heart frequency and respiratory rate. This study was deliberately conducted under conditions as identical as possible to the situation critically ill patients experience in order to reduce the bias on results in future studies (Fig. 2).

The stimulation consisted of three 2D nature videos, each five minutes in length, played as side-by-side videos (SBS videos, scene filmed from only one point of view) in the HMD. We have chosen 2D videos instead of 3D videos since some scenes in 3D could potentially provoke fear (e.g. animal walks towards the user) or elicit vertigo (e.g. view from a mountain) in participants.

All three videos contained natural scenes that were chosen to be enjoyable and cheerful with a visual target (yellow smiley) that was shown at the beginning, the middle, and the end of each video sequence (videos available on our website: see URL). The 2D visual target with a visual angle of 4 degree was superimposed onto the centre of each video frame during the target presentation period. Participants were instructed to fixate the visual target when it was presented. The first video about landscape was filmed from a hot-air balloon perspective (i.e. mountains, meadow, jungle), the second video about water worlds were video scenes filmed either over (i.e. waves on a beach, waterfall in the jungle) or under sea level (coral reef and fishes) and the third video about nature were scenes about wild animals (i.e. panda bears, dolphins, birds). A combination of classic, pop, and orchestral music adapted to the video content was used for auditory stimulation. The music was played via noise-cancelling overhead headphones (Beats Studio by Dr. Dre, Beats Electronics, Santa Monica, USA) to cancel out noise in the ICU station.

The Oculus Rift DK2 (Oculus VR LLC, Irvine, USA) is a head mounted display (HMD) with a resolution of 1920 × 1080 pixels and a frame rate of 90 Hz. Measures taken to reduce cybersickness were a powerful computer (graphic card NVIDIA GTX980, Nvidia, Santa Clara, USA and a CPU Intel i7, Intel, Santa Clara, USA) to prevent low framerates and screen flickering^54^ and positioning participants in horizontal position. To play the videos, the VLC Media Player was used in combination with the Virtual Desktop application developed for the Oculus Rift. To measure gaze behaviour, the SMI Oculus eye tracking system (SensoMotoric Instruments, Teltow, Germany) fitted inside the housing of the Oculus HMD was used. Using image processing, the eye tracking system reconstructs a grayscale image of the eye using six infrared-emitting diodes around each eye. The SMI eye tracker had a sampling frequency of 60 Hz and a system accuracy of 0.5-1 degrees visual angle. To monitor the eyes and physical well-being of the participant, the grayscale images of the eyes were live-streamed to a tablet (iPad Pro, Apple, Cupertino, USA) and monitored by the study investigator. To record whenever a participant fell asleep during stimulation, the eye image stream was stored as video files at a frequency of 30 Hz. Patients which fell asleep were not awakened until the end of the video. Furthermore, all sleep phases were removed before analysing the data.

The respiratory rate and heart frequency were sampled using a five-lead electrocardiography configuration. Using pulse oximetry on a finger, the peripheral capillary oxygen saturation was measured, whereas the mean arterial pressure was measured by a cuff based blood pressure monitor. All vital parameters were stored every second minute and approximated to three measurements in each video (begin, middle, end). The vital parameters were sampled before and during the whole study including recovery phase.

The whole setup was checked by the University Hospital of Berne (Inselspital) and certified to as secure to use in the ICU for healthy participants as well for critically ill patients. All vital parameters were measured with existing and certified devices in the ICU. To prevent feedback into the power supply system and subsequent harm to other ICU devices and patients, a galvanic isolation (Thalheimer Transformers GmbH, Thalheim, Germany) was installed between all net-powered devices. An additional safety measure was the location of the computer 1.5 m away from the participant to guarantee that no current could be transmitted to participants. To prevent the system from dispersing dust and thus putting patients with lowered immune system functioning at risk, all computer fans were protected with a filter. Owing to the direct contact of the HMD and the headphones to the skin of the participants, disposable covers were used for the headphones, while a disinfectable cover made of polyurethane-coated polyester (Obatex bravo, OBA AG, Basel, Switzerland) was tailored for the HMD. The cover and the device were disinfected before and after each participant.

Overall the questionnaire (Table 3) was used to measure how well the VR setup was accepted by the participants and if the stimulation did provoke any discomfort, whereas the psychophysiological and oculomotor data measured the response of the parasympathetic system and the level of attention.

**Table 3:** Questionnaire to measure immersion, presence, usability and sickness in participants.

Nr.	Question (anchors)	Source
1	In the virtual world I had a sense of “being there”. (Not at all - Very much)	IPQ
2	Somehow I felt that the virtual world surrounded me. (Fully disagree - Fully agree)	IPQ
3	How aware were you of the real world surrounding while being in the virtual world? (i.e. sounds, room temperature, other people, etc.)? (Extremely aware - Not aware at all)	IPQ
4	I still paid attention to the real environment. (Fully disagree - Fully agree)	IPQ
5	How real did the virtual world seem to you? (About as real as an imagined world - Indistinguishable from the real world)	IPQ
6	How much did the music in the virtual world involve you? (Not at all - Very much)	PQ
7	I thought the system was easy to use (Fully disagree - Fully agree)	SUS
8	I think that I would like to use this system frequently (Fully disagree - Fully agree)	SUS
9	I felt very confident using the system (Fully disagree - Fully agree)	SUS
10	General discomfort (None - Severe)	SSQ
11	Stomach awareness (None - Severe)	SSQ
12	Sweating (None - Severe)	SSQ
13	Headache (None - Severe)	SSQ
14	Eye strain (None - Severe)	SSQ
15	Nausea (None - Severe)	SSQ
16	Dizziness (None - Severe)	SSQ
17	Which video did you like most?	—

(IPQ: Igroup Presence Questionnaire, PQ: Presence Questionnaire^60^, SUS: System Usability Scale, SSQ: Simulator Sickness Questionnaire).

To measure cybersickness questions from the Simulator Sickness Questionnaire were used^55^. Cybersickness should not be confounded with motion sickness and does occur in all people. In the case of cybersickness, the user is immobile (e.g. standing) but experiences self-motion inside the VR scene. Self-motion is the feeling when the observers’ avatar is moving in the virtual world, while the actual body does not. The symptoms of motion and cybersickness are very similar in nature and include headache, pallor, sweating, and vertigo^56^. The two concepts about immersion (sense of being in the VR world) and presence (how a person reacts to immersion) was assessed by questions out of the Igroup Presence Questionnaire^57^. The higher the immersion and presence, the less a person can distinguish between the real and the virtual world. Thus, immersion and presence are related to technology and can also be described as how closely the experience reflects the real world. Furthermore, it has recently been shown that the higher the sense of presence is, the lower is the risk of experiencing cybersickness^58^. Finally, to assess participants opinion, attitude and perception of the VR system questions out of the System Usability Scale^59^ were used. The questionnaire items had a five-point rating scale between one and five.

### Procedure

First, the participants were instructed and prepared for the study (lying on the bed, fixation of the HMD, headphones, and vital sensors). The preparation phase was followed by a recovery phase of 10 minutes. Before and after the recovery phase, vital parameters were measured once. After the recovery phase, the vital parameters were monitored as two minute medians (median of the sampled vital parameters of the last two minutes) until the end of the study. The stimulation with videos started with a small break between videos after the recovery phase. Finally, after the stimulation participants were asked to answer the questionnaire questions.

### Data pre-processing

To obtain data for analysis, the eye tracking raw data was quantified as a collection of fixation points (gaze points). Fixations were defined as eye movements with minimal duration of 100 ms and a maximal dispersion of two degrees^61^. To account for the small variability of tracking frequency, the gaze points were either up- or down-sampled to a frequency of 60 Hz. Next, image frames in which the participants had their eyes closed were excluded from the analysis. For this, a two-step noise cancelling, followed by canny edge detection (binary image) and a morphological operation was applied. Pupil detection was achieved with a Hough transformation from the pre-processed binary image. The noise cancelling included a Gaussian filter ($\sigma =0.5$) and a median filter of size 8 × 8 pixels. By morphological operation after edge detection, objects containing less than 50 pixels were removed. Eye tracking data was removed when participants closed their eyes for more than 20 frames (0.33 s). At the final stage after pre-processing a sliding window based on a length of 60 s (n−30s and n + 30 s) at a step size of one time unit (1/60 s) was used^62^ for feature extraction (fixation duration [ms/s], number of fixations per second, saccade amplitudes [deg.] and fixation/saccade ratios).

### Statistical analysis

To analyse oculomotor and vital data linear mixed effect models (LMEM), fit by maximum likelihood were used instead of classical linear models or repeated measure ANOVA, since data was clustered and contained numeric values. Conditional F-tests were used to test global fixed effects of categorical variables, conditional t-tests were used for fixed effect parameters and likelihood ratio tests were used for random effects.

First, to examine whether the VR stimulation had a relaxing effect (decreasing of vital signs during stimulation), a LMEM (model 1) with vital sign variables as dependent variables, time and video type as fixed effects, a random intercept for each participant as random effect was fitted and tested for a negative fixed effect of time. To examine whether there were inter-individual differences in the effect of time, a random slope of time per participant was added as random effect to the LMEM (model 2) and tested for variance of the random slopes. To further check whether the relaxing effect was higher for participants with higher baseline vital sign measurements, the correlation between the random intercepts and slopes of time was included in the LMEM (model 3). For each dependent variable, the optimal mixed-effect model was chosen based on the significance tests. In a final explorative step, fixed effects for participant-based ratings of the VR stimulation were added to the respective model in order to investigate the influence of participants experience with the VR environment on vital sign variables by using Holm adjusted p-values.

Secondly, we fitted LMEMs with oculomotor data as dependent variables, fixed effects and a random intercept for each participant as random effect. In order to investigate the relevant predictors, effect sizes were used instead of significance tests, as proposed by Nakagawa *et al*. and Nuthmann *et al*. since the sample size of the oculomotor data was extremely large. The effect size of a dependent variable was calculated by evaluating the goodness of fit of LMEMs by using the marginal (${R}_{{LMM}(m)}^{2}$) and conditional (${R}_{{LMM}(c)}^{2}$) R^2^ ^36–38^. Altogether, we tested eight LMEMs: a zero model without any fixed effects, a full model including all fixed effects (time, target and video type), three individual models for each fixed effect, and three models to quantify the increase of the explained variance by time, target and video type. (Full model without the considered predictor variable). The target was a dummy variable with values zero (not target present) or one (target present). The questionnaire ratings of the VR stimulation were analysed using one-sided t-tests to check whether a significant majority was above the midpoint of the rating scale (2.5) or not.

In the result section only the main findings were discussed, whereas more details about the different LMEMs can be found in the supplementary section. The analysis of the eye tracking and vital data was conducted using MATLAB 2015a^63^ and R for statistics^64^.

### URL

Videos that were used in the VR stimulation are available online on our website http://www.artorg.unibe.ch/research/ger/group_members/persons/gerber_stephan/index_eng.html#pane482606.

### Data and Code availability

All relevant data and codes supporting the findings are presented in this paper and in the supplementary information. Further data and code of the study are available upon request.

## Electronic supplementary material

Supplementary Results
