# Supplementary material for: Visuo-acoustic stimulation that helps you to relax: A virtual reality setup for patients in the intensive care unit
Source: Sci Rep. 2017 Oct 16;7:13228. doi: 10.1038/s41598-017-13153-1 (PMC5643433; doi:10.1038/s41598-017-13153-1)
Supplement: Supplementary file 1 — Supplementary Results [file 41598_2017_13153_MOESM1_ESM.pdf]

Visuo-acoustic stimulation that helps you to relax: A virtual reality setup for patients in the intensive care unit.

Stephan Gerber[1,#], Marie-Madlen Jeitziner[2,#], Patric Wyss[1], Alvin Chesham[1], Prabitha Urwyler[1,3], René M. Müri[1,4], Stephan M. Jakob[2,+] & Tobias Nef[1,5,+,*]

[1] Gerontechnology & Rehabilitation Group, University of Bern
[2] Department of Intensive Care Medicine, University Hospital Bern (Inselspital), University of Bern
[3] University Hospital of Old Age Psychiatry, University of Bern
[4] Department of Neurology, University Neurorehabilitation, University Hospital Bern (Inselspital), University of Bern
[5] ARTORG Center for Biomedical Engineering Research, University of Bern

#) equal contribution (shared first authorship)

+) equal contribution (shared last authorship)

[*] E-mail: tobias.nef@artorg.unibe.ch

Supplementary Results

Effect of time, age and ratings in vital signs measurements

The comparison between the second (without correlated random effects) and the first model revealed a highly significant variance of the random slopes (χ^2 (1) = 49.84, p < 0.001) in HF, whereas no significant correlation between the random effect could be found when comparing model 3 and model 2 (χ^2 (1) = 2.72, p = 0.10). The same was true for SpO_2 where the variance of the random slopes (χ^2 (1) = 49.33, p < 0.001) was significant and the correlation between the random effects (χ^2 (1) = 2.97, p = 0.085) was not. In case of the RF both likelihood ratio tests (model 2 vs. model 1: χ^2 (1) = 87.18, p <0.001 and model 3 vs. model 2: χ^2 (1) = 44.12, p <0.001) revealed a highly significant result, the same belongs to MAP (model 2 vs. model 1: χ^2 (1) = 6.39, p = 0.012 and model 3 vs. model 2 χ^2 (1) = 8.31, p = 0.004)). Therefore, based on the results of the tests, we selected model 2 for HF and SpO_2 and model 3 for RF and MAP for the further analysis.

The fixed time effect as seen in figure 1 of the selected models was significant for HF (t (277) = -2.704, p = 0.007), RF (t (277) = -2.02, p = 0.044) and MAP (t (277) = -1.98, p = 0.049) but not for SpO_2 (t (277) = -1.38, p = 0.170).

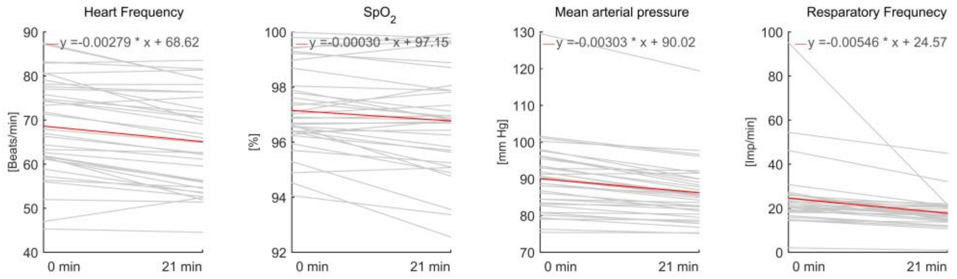

Figure 1| Selected LMEMs to analyze the fixed time effect of the vital signs measurements. For HF and SpO_2 model 2 was used. Model 2 includes time and video type as fixed effects and a random intercept and slope for all participants. In case of RF and MAP model 3 was used. In addition to model 2 it also included a random slope, but with correlated random effects.

The fixed interaction effect between time and age was not significant for the vital parameters HF (t (276) = -0.963, p = 0.337), SpO_2 (t (276) = -1.128, p = 0.260) and RF (t (278) = -0.309, p = 0.757), except for MAP (t (276) = -2.149, p = 0.033. For HF (t (33) = -0.914, p = 0.367) and RF (t (33) = -0.425, p = 0.674) the fixed age effect did not reach significance, while for SpO_2 the fixed age effect was (t (33) = -2.759, p = 0.009).

Second, explorative analyses further including questionnaire ratings of the videos and the stimulation (immersion, spatial presence, involvement, realism, usability, nausea, oculomotor problems and disorientation), a fixed interaction between time and age in MAP, and a fixed effect for age in SpO_2 were performed. Using a significance level of 5% (p-val) per test (t (26) = 2.857, p =0.0083), a significant fixed effect for nausea was found in RF (t (33) = 2.857, p = 0.0.0083). However, with a global significance level (Holm-adjusted p-values, p-adj) of 5% for all tests with regard to the questionnaire, the fixed effect of nausea was no longer significant (table 1).

Table 1 | Explorative search of additional fixed effects in vital parameters

	HF			SpO_2		
	t-val (26)	*p-val*	*p-adj*	*t-val (25)*	*p-val*	*p-adj*
Immersion	0.174	0.863	1.000	-1.283	0.211	1.000
Spatial presence	0.175	0.863	1.000	-0.134	0.894	1.000
Involvement	0.884	0.385	1.000	-1.178	0.250	1.000
Realism	0.542	0.592	1.000	0.936	0.358	1.000
Usability	0.572	0.572	1.000	0.227	0.822	1.000
Nausea	0.651	0.521	1.000	0.939	0.357	1.000
Oculomotor problems	-1.970	0.060	0.477	-0.247	0.807	1.000
Disorientation	0.539	0.595	1.000	0.285	0.778	1.000

	RF			MAP		
	t-val (26)	*p-val*	*p-adj*	*t-val (25)*	*p-val*	*p-adj*
Immersion	-0.0355	0.972	1.000	0.0173	0.986	1.000
Spatial presence	0.611	0.547	1.000	-0.958	0.347	1.000
Involvement	-0.712	0.483	1.000	-0.223	0.826	1.000
Realism	0.413	0.683	1.000	-0.476	0.638	1.000
Usability	-1.397	0.174	1.000	-0.835	0.412	1.000
Nausea	2.857	0.008	0.067	-0.0893	0.930	1.000
Oculomotor problems	0.298	0.768	1.000	-1.110	0.278	1.000
Disorientation	-0.536	0.596	1.000	-0.194	0.848	1.000

Effect of time, target in oculomotor data

The variance explained by the fixed effects ($R^2_{LMM(m)}$) of the full model was highest for number of fixations 32.2 % and lowest for fixation/saccade ratio 9.0 %. The same is true for the variance explained by the fixed and random effects ($R^2_{LMM(c)}$), which was 71.1 % for number of fixations and 36.8 % for the fixation/saccade ratio.

The fixed effect of video type ($R^2_{LMM(m)}$, 21.3 %) explained most of the variance for number of fixations. On the other hand, for fixation duration, saccade amplitude and fixation/saccade ratio the goodness of fit of the fixed effect was highest in the model including the target (Min = 6.5 %, Max = 12.64 %) and lowest for time (Min = 1.2 %, Max = 5.5 %). The additional increase of variance explained by time (full model compared to a model with fixed effects for video type and the considered variable) was negligible for both marginal (Min = 0.044%, Max = 0.28 %) and conational R^2 (Min = 0.009 %, Max = 0.28 %). The highest impact was found for the fixed effect target, which increased the variance between $R^2_{LMM(m)}$ Min = 6.5 %, Max = 12.6 % and $R^2_{LMM(c)}$ Min = 6.6 %, Max = 12.8 %.

For video type, the increase of variance was only relevant for the number of fixations ($R^2_{LMM(m)}$ = 6.5 % and $R^2_{LMM(c)}$ = 7.9 %), whereas for fixation duration, saccade amplitude and fixation/saccade ratio was not. Further findings can be seen in table 2.

Table 2 | Marginal and conditional R^2 in oculomotor data

Model*	Nr. of fixations		Fixation duration		Saccade Amplitude		Fixation/Saccade ratio	
	$R^2_{LMM(m)}$	$R^2_{LMM(c)}$	$R^2_{LMM(m)}$	$R^2_{LMM(c)}$	$R^2_{LMM(m)}$	$R^2_{LMM(c)}$	$R^2_{LMM(m)}$	$R^2_{LMM(c)}$
1 - Zero	0	40.150	0	33.594	0	37.270	0	27.568
2 - Time	14.920	52.167	3.769	35.729	5.541	41.048	1.241	27.860
3 - Target	10.657	50.897	12.640	46.404	8.865	46.361	6.517	34.203
4 - Video type	21.269	59.908	5.553	38.521	6.031	41.865	2.423	29.919
5 - Full	32.322	71.081	18.348	51.597	15.044	51.09	9.040	36.773
Increase of time	0.276	0.284	0.0998	0.184	0.0443	0.00879	0.111	0.213
Increase of target	10.713	10.821	12.628	12.799	8.950	9.207	6.462	6.582
Increase of video	6.547	7.944	1.782	2.878	0.558	0.844	1.255	2.228

* All models had a random intercept per subject. The fractional models 2-4 included one of the fixed effects in addition to model 1, whereas the full model (model 5) contained all fixed effects (i.e. time, target and video type). Increase: full model compared to model without fixed effect of the considered variable.
